# The outcome of surgical correction of severe scoliotic spinal deformities on sagittal balance in adolescents

**DOI:** 10.3389/fped.2026.1830989

**Published:** 2026-07-08

**Authors:** Nurbek Nadirov, Sergey Vissarionov, Dmitriy Kokushin, Arman Batpen, Marat Asadulaev, Murat Baidarbekov, Dina Saginova, Magauiya Nurislam

**Affiliations:** 1 Department of Orthopedics, Mother and Child Health Center, University Medical Center, Astana, Kazakhstan; 2 H.Turner National Medical Research Center for Children's Orthopedics and Trauma Surgery of the Ministry of Health of the Russian Federation, Saint Petersburg, Russia; 3 National Scientific Center of Traumatology and Orthopedics Named After Academician N. D. Batpenov, Astana, Kazakhstan

**Keywords:** children, idiopathic scoliosis, lumbar lordosis, sagittal balance, surgical treatment, thoracic kyphosis, treatment results

## Abstract

**Introduction:**

In recent years, the concept of sagittal trunk balance in patients with spinal deformity has been significantly developed. The main disorders in the sagittal plane are decreased thoracic kyphosis, compensatory decrease in lumbar and cervical lordosis, and negative sagittal balance. However, these issues remain insufficiently studied in the literature.

**Objective:**

To show, analyze and clarify the effect of surgical correction of spinal deformity in adolescents with severe forms of idiopathic thoracic scoliosis on the parameters of sagittal balance, outcomes and efficiency of surgical treatment for sagittal balance.

**Materials and methods:**

Patients with idiopathic thoracic scoliosis (Lenke types 1–3) aged 12–17 years underwent discapophysectomy at the apex of the main curve, intraoperative halo traction, and posterior multi-support metal fusion with stabilization in one session. All operations were performed at the G.I. Turner National Research Medical Center between 2017 and 2025.

**Results:**

There were 5 boys (13.2%), 33 girls (86.8%) with average age of the patients was 15.0 ± 1.3 years. Main thoracic curves ranged from 80° to 140° according to Cobb (average—102.8°). A positive correlation of moderate strength was revealed between the values of TK and SVA (Spearman correlation coefficient 0.33, *p* < 0.05), a negative correlation of moderate strength between the values of TK and SSA (Spearman correlation coefficient −0.31, *p* < 0.05).

**Conclusion:**

Discapophysectomy at the apex of the main curvature, intraoperative halo traction, and correction of deformity with a posterior multi-support metal spinal fusion and stabilization in one surgical session contributed to achieving normal global and regional parameters of sagittal balance in adolescent patients with severe forms of idiopathic thoracic scoliosis.

## Introduction

### Significance of the study

In recent years, the concept of sagittal trunk balance in patients with spinal deformity of various localization has received significant development (1). The results of scientific research indicate that achieving close to physiological parameters of sagittal balance as a result of surgical treatment was crucial to ensure optimal functional results and preserve results in the long-term postoperative period (2).

The main part of the publications devoted to the study of the features of sagittal balance in patients with idiopathic scoliosis includes patients with spinal column deformity in the frontal plane of no more than 80° according to Cobb (3, 4). The main disorders in the sagittal plane in patients of this group are a decrease in the values of thoracic kyphosis (TK) and the compensatory mechanisms accompanying this pathological condition—a decrease in the values of lumbar and cervical lordosis, the development of a negative sagittal balance of the trunk. These changes are recorded both before and after surgical correction of spinal deformity (5). At the same time, it should be noted that patients with severe forms of idiopathic scoliosis have the paradoxical kyphosis described by J. Dubousset, which develops as a result of axial rotation of the vertebrae up to 90° and their lateral collapse (6). Based on a number of scientific studies, such changes are characteristic of patients with a magnitude of the main deformity of more than 100° according to Cobb and it accompanied by opposite compensation mechanisms (7).

Despite significant changes of the regional parameters of the sagittal balance of the trunk, deviations in the values of global parameters in patients of this group are rarely recorded due to the possibilities of compensatory mechanisms (7). The main mechanisms for compensating for increased TK values in young patients are overextension in adjacent parts of the spine—lumbar and cervical. When these compensatory capabilities are limited, pelvic retroversion develops and a further cascade of compensatory mechanisms observed and aimed at eliminating the anterior imbalance (8).

Surgical correction of severe scoliotic spinal deformities, including extensive spinal fusion, inevitably leads to changes in the parameters of the sagittal balance of the trunk and limited compensatory capabilities (5). However, the presented problems in the scientific literature are reflected in isolated publications, which require further study and analysis.

### The aim of the study

To analyze the effect of the results of surgical correction of spinal deformity in adolescents with severe forms of idiopathic thoracic scoliosis on the parameters of sagittal balance.

## Materials and methods

### Study design

The study design is a single-center cohort retrospective study. The study included 38 patients with idiopathic scoliosis aged 12–17 years who received surgical treatment at the G.I. Turner National Research Medical Center for Pediatric Traumatology and Orthopedics of the Ministry of Health of the Russian Federation in the period from 2017 to 2025.

### Surgical technique

All patients underwent surgical intervention in the scope of discapophysectomy at the apex of the main curvature, intraoperative halo traction, correction of the deformity with a posterior multi-support metal fusion and stabilization in one surgical session.

### Inclusion and exclusion criteria

#### Inclusion criteria

Idiopathic scoliosis with a main curvature in the thoracic spine (type 1, 2 and 3 according to Lenke classification). Age of patients from 12 to 17 years inclusive. Panoramic radiographs of the spine in 2 projections performed in the standing position before and after surgical correction of spinal deformity. The modification of the main arch of scoliotic deformity in the frontal plane was more than 80° deg according to Cobb.

#### Exclusion criteria

Patients with congenital pathology of the spine, spinal canal and spinal cord, as well as systemic pathology of the musculoskeletal system; orthopedic pathology of the lower extremities; previous operations on the spine, pelvic ring bones and chest; radiographs of the spine of poor quality, which do not reliably calculate the parameters of the sagittal balance of the trunk, patients who refused to participate in the study.

### Radiographic assessment

All patients underwent panoramic radiographs of the spine in straight and lateral projections in the standing position before surgery and 10–14 days after surgery. The x-ray images obtained were analyzed using the Surgimap 2.3.2.1 program. Radiometric calculations of the following parameters were performed: the rate of the main curvature of scoliotic deformity in the thoracic spine, thoracic kyphosis Th4-Th12 (thoracic kyphosis—TK) and lumbar lordosis L1-S1 (lumbar lordosis—LL) using the Cobb angles, pelvic angle (pelvic incident — PI), sacral tilt angle slope — SS), pelvic tilt angle (pelvic tilt — PT), the difference between the values of the pelvic index and lumbar lordosis (PI-LL), the values of the sagittal vertical axis (SVA), the vertebral sacral angle (spino-sacral angle — SSA), TPA (T1/pelvic angle).

### Statistical analysis

Statistical analysis performed using the program Jamovi 2.7.5. Arithmetic averages (M), standard deviations (SD), median (Me) with an interquartile range (IQR) were calculated, and minimum (Min) and maximum (Max) values were determined. The normality of the data distribution checked using the Shapiro–Wilk test. To perform a comparative analysis of the parameters of the sagittal balance before and after surgical treatment, nonparametric tests for dependent samples were used—the Wilcoxon criterion for quantitative and the McNemar test for qualitative data. Correlation analysis was performed using Spearman's criterion, while the strength of the bond determined by the following indicators: 0.01 ≤ *p* < 0.29 — weak bond; 0.30 ≤ *p* < 0.69 — moderate bond; 0.70 ≤ *p* < 1.00 — strong bond. The null hypothesis in statistical tests rejected at a significance level of *p* < 0.05.

### Main part

**Figure 1:**
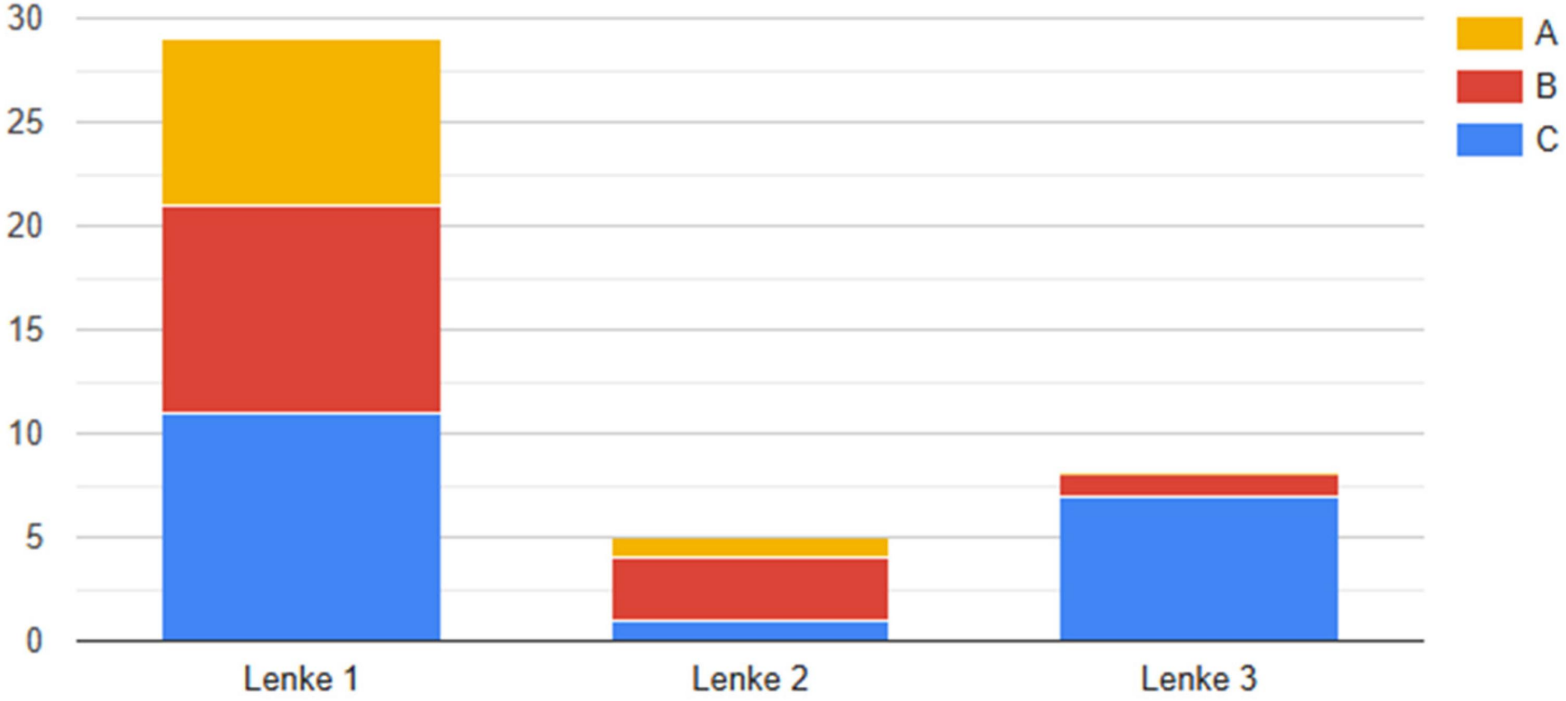
The distribution of patients by types of deformity, according to the lenke classification: A, B, C — lumbar modifiers according to the lenke classification.

The study included 38 patients with idiopathic thoracic scoliosis aged 12–17 years with a major deformity arch from 80° to 140° according to Cobb (average—102.80). Of these, there were 5 (13.2%) boys, 33 (86.8%) girls. The mean age of the patients was 15.0 ± 1.3 years. The distribution of deformations according to the Lenke classification is shown in Figure 1. All patients underwent surgical intervention aimed at correcting scoliotic spinal deformity to the specified extent.

**Figure 2:**
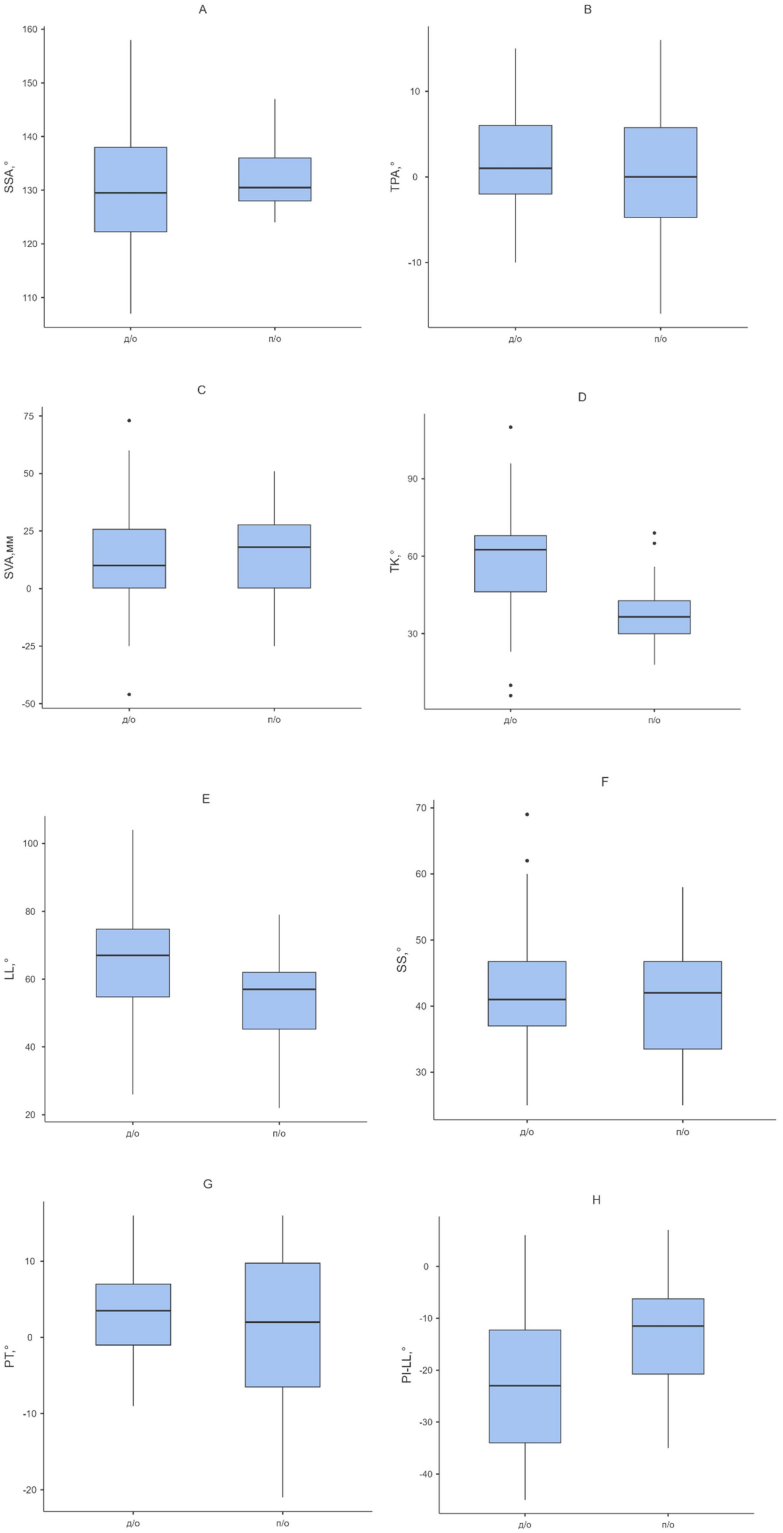
Changes in sagittal balance parameters in patients with severe forms of idiopathic thoracic scoliosis after surgical correction of spinal deformity. SSA, spino-sacrale angle; TPA, T1/pelvic angle; SVA, sagittal vertical axis; TK, thoracic kyphosis; LL, lumbar lordosis; SS, sacral scope; PT, pelvic tilt; PI–LL, the difference between the values of the indicators: pelvic incidence and lumbar lordosis.

The results of a descriptive and comparative statistical analysis of the previously indicated radiological parameters in patients included in the study are presented in Table 1 and Figure 2.

**Table 1:** Outcomes of x-ray examination of patients and comparative data analysis of the studied parameters before and after surgical treatment.

Indicators	Before and after operation	M ± SD	Ме (IQR)	Min	Max	Shapiro–Wilk test, p	Wilcoxon test, p	Regulatory indicators in healthy adolescents according to literature data (M ± SD) (15,16)
Main curvature,°	b/о	102.8 ± 16.5	104.0 (28.8)	80.0	140.0	0.061	<.001	-
	a/о	37.5 ± 12.5	36.5 (11.8)	11.0	68.0	0.195		
SSA,°	b/о	130.6 ± 10.6	129.5 (15.8)	107.0	158.0	0.805	0.256	132.7 ± 8.0
	a/о	132.0 ± 5.5	130.5 (8.0)	124.0	147.0	0.036		
TPA,°	b/о	2.3 ± 5.8	1.0 (8.0)	−10.0	15.0	0.343	0.632	<14.0^a^
	a/о	0.4 ± 8.0	0.0 (10.5)	−16.0	16.0	0.784		
SVA, мм	b/о	13.5 ± 24.0	10.0 (25.5)	−46.0	73.0	0.181	0.306	−9.0 ± 44
	a/о	14.3 ± 18.8	18.0 (27.5)	−25.0	51.0	0.066		
TK,°	b/о	58.8 ± 23.7	62.5 (21.8)	6.0	110.0	0.261	<.001	45.8 ± 10.4
	a/о	37.2 ± 12.0	36.5 (12.8)	18.0	69.0	0.154		
LL,°	b/о	65.8 ± 16.7	67.0 (20.0)	26.0	104.0	0.907	<.001	57.7 ± 11.1
	a/о	54.0 ± 12.4	57.0 (16.8)	22.0	79.0	0.475		
SS,°	b/о	41.3 ± 10.3	41.0 (9.8)	25.0	69.0	0.109	0.933	39.1 ± 7.6
	a/о	40.7 ± 8.4	42.0 (13.3)	25.0	58.0	0.603		
PT,°	b/о	3.1 ± 6.1	3.5 (8.0)	−9.0	16.0	0.736	0.085	7.7 ± 8.3
	a/о	1.3 ± 9.6	2.0 (16.3)	−21.0	16.0	0.184		
PI-LL,°	b/о	−21.4 ± 13.4	−23.0 (21.8)	−45.0	6.0	0.290	0.002	<9.0^a^
	a/о	−13.2 ± 9.9	−11.5 (14.5)	−35.0	7.0	0.142		

SSA, spino-sacral angle; TPA, T1/pelvic angle; SVA, sagittal vertical axis; TK, thoracic kyphosis; LL, lumbar lordosis; PI, pelvic inclination; SS, sacral scope; PT, pelvic tilt; PI–LL, the difference between the values of the indicators: pelvic incidence and lumbar lorodsis; b/о, before operation; a/о, after operation.

a the scientific literature does not describe the normative indicators of these parameters in adolescent patients, but the target values for adults are indicated;.

**Figure 3:**
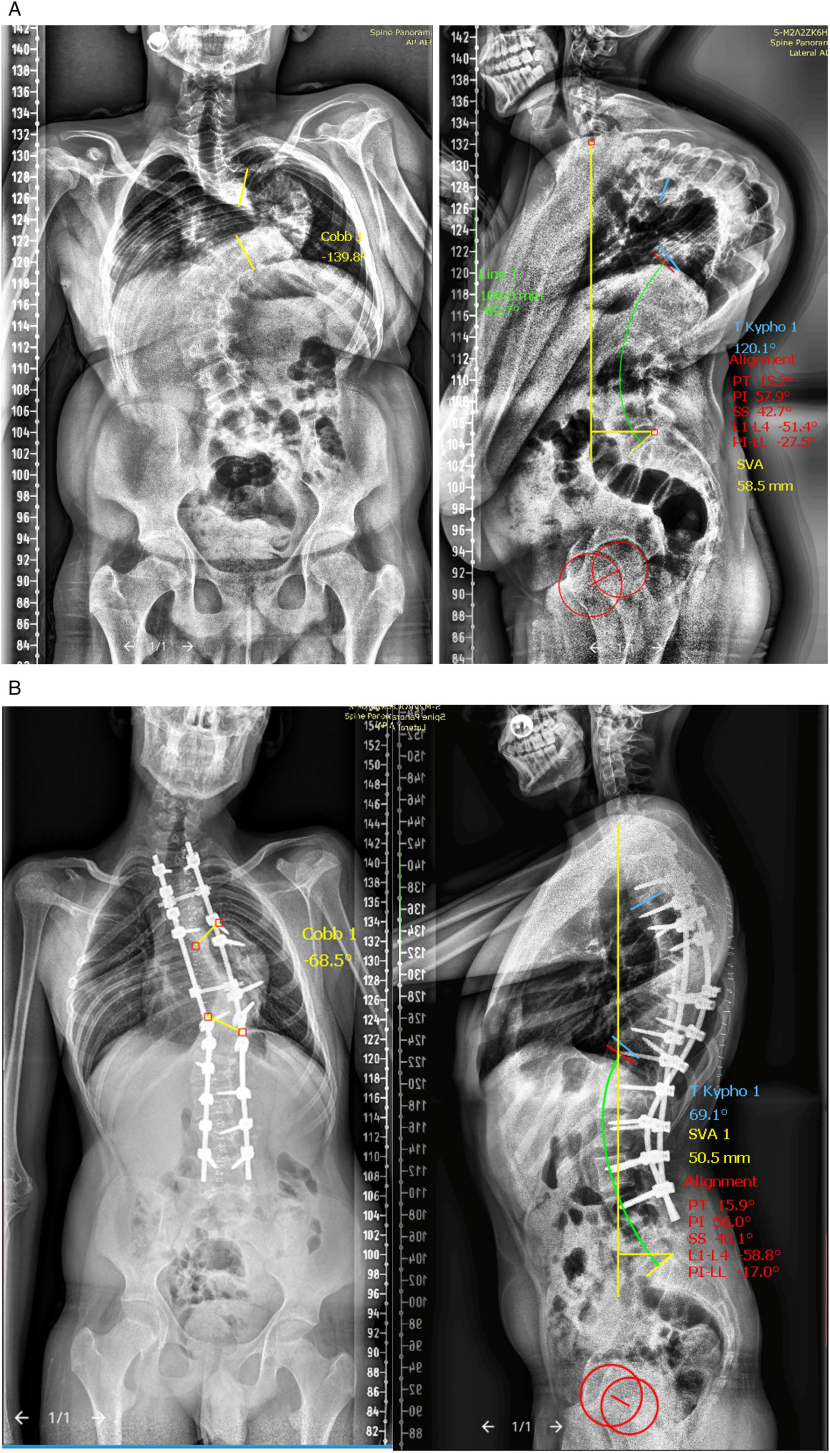
**(A)** before operation. A clinical example of a patient T., 17 years old. Diagnosis: Idiopathic left-sided thoracic scoliosis, grade IV. **(B)** after operation. A clinical example of a patient T., 17 years old. Diagnosis: Idiopathic left-sided thoracic scoliosis, grade IV.

Deviation of global sagittal balance parameters observed in 11 (28.9%) of 38 patients before surgery (SSA, TPA, SVA). A decrease in the value SSA <125° noted in 11 patients, in 6 patients SVA magnification >35 mm, in 1 patient the increase in TPA >14°. At the same time, no deviation of PT >16° and PI-LL >9° parameters registered in any of the patients. After performing surgical intervention aimed at correction of scoliotic spinal deformity, the above-described disorders were registered in only 2 (5.3%) patients. One of them had a deviation of all three parameters of the global sagittal balance of the trunk (Figure 3, Table 2), the second patient has only a deviation of the SVA values. When comparing the frequency of x-ray signs of anterior imbalance in patients before and after surgical treatment, there was a statistically significant difference (McNemar's test, *p* < 0.001).

**Table 2:** Results of x-ray examination of patient T. 17 years old before and after surgery.

Before and after operation	Main curveture,°	SSA,°	TPA,°	SVA,°	TK,°	LL,°	SS,°	PT,°	PI-LL,°
b/о	140.0	122.0	15.0	59.0	120.1	85.5	42.7	15.2	−27.5
a/о	68.5	124.0	16.0	50.5	69.1	73.0	40.1	15.9	−17.0

b/о, before operation; a/о, after operation.

All 2 patients with a deviation in the parameters of global sagittal balance after surgical treatment had an excess of physiological values TK >60° by Cobb. These patients also had the highest values of deformity in the frontal plane and TK during preoperative examination among all patients included in the study. A positive correlation of moderate strength revealed between the values of TK and SVA (Spearman correlation coefficient 0.33, *p* < 0.05), a negative correlation of moderate strength between the values of TK and SSA (Spearman correlation coefficient −0.31, *p* < 0.05).

## Discussion

When assessing the global parameters of sagittal balance (SSA, TPA, SVA), no statistically significant difference found between the quantitative data in patients before and after surgery (Table 1, Figures 2A–C). However, x-ray signs of anterior imbalance development in the postoperative period were recorded significantly less frequently in patients (statistically significant difference, McNemar test, *p* < 0.001). A significant risk factor for maintaining anterior imbalance in patients with severe forms of idiopathic scoliosis after surgical treatment, according to the analysis, was insufficient correction of the kyphotic component of the deformity. In all 2 patients with radiological signs of anterior imbalance after surgery, TK values exceeded physiological values (>60° Cobb). A clinical example is shown in Figure 3. The results of the x-ray examination of this patient are shown in Table 2. Other researchers come to similar conclusions in their study (9).

Surgical intervention in all patients performed in the amount of discapophysectomy at the top of the deformity in combination with corporodesis, intraoperative halo traction, correction of the curvature of the posterior multi-support metal fusion and stabilization of the achieved result in one surgical session. Carrying out a certain sequence of corrective manipulations using transpedicular spinal systems, in addition to ensuring optimal correction of deformity in the frontal plane, made it possible to achieve global and regional sagittal balance parameters close to physiological values in adolescent patients with severe forms of idiopathic scoliosis in most cases. However, in the case of superheavy deformations (>120–130° Cobb), which are characterized by the presence of a pronounced kyphotic component, the corrective capabilities of this technology may not be sufficient.

The persistent imbalance after surgical correction of spinal deformity in patients with idiopathic scoliosis can lead to accelerated development of degenerative processes in the spinal motor segments not included in the fusion zone, the development of pain syndrome and a decrease in quality of life (1). In the study by P. Bernstein et al. lumbar MRI was performed in patients with idiopathic scoliosis 5–10 years after surgical correction of spinal deformity. The deviation of the sagittal balance parameters influenced the level of hydration of the intervertebral discs to a greater extent than the choice of the lower instrumented vertebra (LIV) (10). Similar results can be found in the work of other researchers (11, 12).

In addition, the available scientific literature actively discusses the relationship between the parameters of the sagittal balance of the trunk and the incidence of PJK and DJK in patients with spinal deformities in the postoperative period (9). However, the development of these complications in patients with idiopathic scoliosis was more often associated with a decrease in TK values (5).

## Discussion and conclusion

Currently, according to the literature, more and more data is fostering on the importance and necessity of analyzing the parameters of sagittal balance in patients with musculoskeletal pathology, including in patients with idiopathic scoliosis (5, 7, 13, 14). Further work in this area is relevant and promising in order to improve the treatment outcomes of this group of patients.

## Limitations of the research

The disadvantages of this study include the small sample size of patients, the lack of long-term treatment results, the results of a clinical examination of patients and an assessment of their quality of life.

## Results

### Patient demographics

In the study, 38 patients were included with idiopathic thoracic scoliosis aged 12–17 years with the main curvature from 80° to 140° according to Cobb (average—102.80). According to the gender, there were 5 boys (13.2%), 33 girls (86.8%). The average age of the patients was 15.0 ± 1.3 years.

### Correction of the coronal deformity

Main thoracic curves ranged from 80° to 140° according to Cobb (average—102.8°). After surgical correction, the main curvature was reduced to 37.5 ± 12.5° (Table 1, Figure 2).

### Correlations

A positive correlation of moderate strength revealed between the values of TK and SVA (Spearman correlation coefficient 0.33, *p* < 0.05), a negative correlation of moderate strength between the values of TK and SSA (Spearman correlation coefficient −0.31, *p* < 0.05).

## Conclusion

Discapophysectomy at the apex of the main deformity, intraoperative halo traction, and correction of deformity with a posterior multi-support metal fusion and stabilization in one surgical session contributed to achieving normal global and regional parameters of sagittal balance in adolescent patients with severe forms of idiopathic thoracic scoliosis. However, in the case of superheavy deformities (>120–130° Cobb), which are characterized by the presence of a pronounced kyphotic component of the curvature, it is necessary to consider the use of additional technologies that can significantly correct thoracic kyphosis. It can help achieve close to physiological parameters of the sagittal balance of the trunk and improve treatment outcomes in this category of patients.

## Data Availability

The original contributions presented in the study are included in the article/Supplementary Material, further inquiries can be directed to the corresponding author.
